# Feasibility and oncological outcomes of video‐assisted thoracic surgery versus thoracotomy for pathologic N2 disease in non–small cell lung cancer: A comprehensive systematic review and meta‐analysis

**DOI:** 10.1111/1759-7714.14614

**Published:** 2022-09-14

**Authors:** Xiaogang Li, Kaili Huang, Hanyu Deng, Qiangqiang Zheng, Tao Xiao, Jinming Yu, Qinghua Zhou

**Affiliations:** ^1^ Lung Cancer Center West China Hospital of Sichuan University Chengdu China; ^2^ Department of Radiation Oncology and Shandong Provincial Key Laboratory of Radiation Oncology Shandong First Medical University and Shandong Academy of Medical Sciences Jinan China; ^3^ Research Unit of Radiation Oncology Chinese Academy of Medical Sciences Jinan China

**Keywords:** non–small cell lung cancer, outcomes, pathologic N2, thoracotomy, VATS

## Abstract

This meta‐analysis aimed to evaluate the feasibility and oncological outcomes between video‐assisted thoracic surgery (VATS) and thoracotomy for non–small cell lung cancer (NSCLC) patients with pathologic N2 (pN2) disease. Data for analysis included short‐term outcomes and long‐term outcomes. We calculated the weighted mean differences (WMDs) for continuous data and the results of overall survival (OS) and disease free survival (DFS) were pooled using the hazard ratios (HRs) with 95% confidence intervals (CIs). Heterogeneity was assessed using the Q‐test and I^2^‐test. Sensitivity analysis was performed to further examine the stability of pooled HRs and WMDs. In the pooled analyses of 10 eligible studies, results showed that VATS for NSCLC patients with pN2 disease yielded significantly less blood loss (WMD = −61.43; 95% confidence intervals [CI], [−87.69, −35.18]; *p* < 0.001), less post‐operation hospital stay (WMD, −1.62; 95% CI, [−2.96, −0.28]; *p* = 0.02), and comparable operation time (WMD,  −8.32; 95% CI, [−23.88, 7.23]; *p* = 0.29), post‐operation complication rate (risk ratio [RR], 0.95; 95% CI, [0.78, 1.15]; *p* = 0.59), chest tube duration to thoracotomy (WMD, −0.64; 95% CI, [−1.45, 0.17]; *p* = 0.12), extent of lymph node dissection (WMD, −1.46; 95% CI, [−3.87, 0.95]; *p* = 0.23) and 1‐year OS (HR, 1.30; 95% CI, [0.96, 1.76]; *p* = 0.09) than thoracotomy. However, VATS may improve 3‐year OS (HR, 1.26; 95% CI, [1.12, 1.42]; *p* = 0.0002) and yield comparable 1‐year DFS (HR, 1.14; 95% CI, [0.89, 1.46]; *p* = 0.32) and 3‐year DFS (HR, 1.03; 95% CI, [0.88, 1.22]; *p* = 0.70) for NSCLC patients with pN2 disease than thoracotomy. VATS could yield less surgical trauma and improve post‐operative recovery than thoracotomy. Moreover, VATS may improve the oncological outcomes of those patients.

## Graphical abstract

VATS for NSCLC patients with pN2 disease yielded comparable 1‐year OS (HR, 1.30; 95% CI, [0.96, 1.76]; *p* = 0.09) as thoracotomy. VATS may improve 3‐year OS (HR, 1.26; 95% CI, [1.12, 1.42]; *p* = 0.0002) for NSCLC patients with pN2 disease compared with thoracotomy.

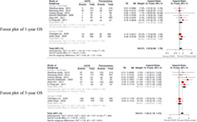

## INTRODUCTION

Lung cancer, mainly composed of small cell lung cancer and non–small cell lung cancer (NSCLC), has become the leading cause of cancer‐related death worldwide.^1^ Although many therapeutic modalities have been applied in clinical practice to improve the survival of patients with NSCLC, surgery still plays an important role in the management of patients with resectable NSCLC, especially for those at early stage.^2^, ^3^, ^4^, ^5^, ^6^ For patients with NSCLC, mediastinal lymph node (MLN) metastasis is one of the most crucial prognostic factors and neoadjuvant therapy is indicated before curative surgery for NSCLC patients with positive ipsilateral mediastinal and/or subcarinal lymph nodes (N2).^7^ Therefore, accurate preoperative clinical node (N) staging is extremely important. However, false negatives may exist in both preoperative non‐invasive workups, such as computed tomography (CT) and positron emission tomography (PET), and invasive workups, such as endobronchial ultrasonography guided transbronchial needle aspiration (EBUS‐TBNA) and mediastinoscopy,^8^, ^9^ resulting in ~ 10% patients with mediastinal lymph node metastasis deemed as candidates for surgery as the first‐line therapy.^10^ Therefore, a clinical dilemma is encountered in those patients, as guidelines advise induction therapy before surgery.^11^ Patients with N2 disease constitute a heterogeneous population, such as patient with an incidental, pathologically identified, single focus of mediastinal disease or patient with clinically identified multilevel bulky unresectable disease, pertaining quite different prognosis.^12^, ^13^ Recent studies suggested that upfront surgery seemed to be feasible for NSCLC patients with resectable (non‐bulky or discernable) N2 diseases.^14^, ^15^

During the past two decades, video‐assisted thoracic surgery (VATS), as a minimally invasive surgical technique, has been widely adopted by thoracic surgeons since it was introduced at the beginning of the 1990s,^16^ and subsequent studies have consistently demonstrated its safety and feasibility versus traditional thoracotomy for early‐stage lung cancer.^17^ However, the risk of incomplete lymph node dissection and the missed opportunity of adjuvant chemotherapy because of unsuspected nodal metastasis during VATS has raised a major concern for its clinical use in pathologic N2 (pN2) patients.^18^ Therefore, it remains controversial whether conversion from VATS to thoracotomy is necessary when intraoperative lymph node (LN) sampling reveals pathologic N2 disease.^19^ Moreover, the feasibility and oncological outcomes of VATS versus thoracotomy for pN2 disease in resectable NSCLC is still under debate.^10^, ^14^, ^20^, ^21^, ^22^, ^23^, ^24^, ^25^, ^26^, ^27^ Previous meta‐analysis was performed to determine oncological outcomes and feasibility of VATS for surgical treatment of pathologic N2 NSCLC and found that no significant differences between VATS and thoracotomy, but there were only 3 papers^10^, ^20^, ^23^ with relative small sample size included for comparison.^19^ However, after carefully searching and evaluation, we found more relevant evidence to provide sufficient analytical power to compare the efficacy of VATS and thoracotomy in treating pN2 NSCLC.^14^, ^21^, ^22^, ^24^, ^25^, ^26^ Therefore, we tried to conduct an up‐to‐date comprehensive meta‐analysis comparing the feasibility and oncological outcomes of VATS versus thoracotomy for pathologic N2 NSCLC. To our knowledge, this is the most comprehensive meta‐analysis with largest sample size focusing on current topic.

## MATERIALS AND METHODS

This review was conducted according to the Preferred Reporting Items for Systematic Reviews and Meta‐analysis (PRISMA) guidelines for systematic reviews,^28^ and ethical approval from the institutional review board or written consent from patients was not needed because individual patient data were not involved.

### Data sources and search strategy

Systematic computerized search of EMBASE, PUBMED, Cochrane library, and Web of Science for studies date up to April 2021, was performed with the following search terms: (open surgery OR thoracotomy) AND (VATS OR video‐assisted thoracoscopic surgery) AND (lung cancer OR lung carcinoma OR lung neoplasms) AND (N2). There were no language or publication restrictions and all reference lists from the studies selected by electronic searching were scanned to further identify relevant studies.

### Study endpoints

The primary endpoints of this meta‐analysis were the long‐term outcomes (1‐year overall survival [OS] and disease‐free survival [DFS] as well as 3‐year OS and DFS) of VATS versus thoracotomy for pN2 NSCLC patients and the second endpoints were short‐term outcomes (operation time, blood loss, complications, chest tube duration, post‐operative hospital stay, and lymph node dissected) between VATS and thoracotomy for pN2 NSCLC patients receiving lobectomy.

### Inclusion and exclusion criteria

We formulated the inclusion criteria to select eligible studies to conduct this meta‐analysis as follows: (1) study focused on NSCLC patients who were clinical N0 before surgery, but found to have mediastinal lymph node metastasis intra‐ or post‐operatively pN2; (2) study compared the effects of VATS lobectomy with thoracotomy lobectomy for pN2 NSCLC; (3) study provided sufficient perioperative and long‐term oncological data for analysis; and (4) study should be either randomized controlled trial (RCT) or observational study. Exclusion criteria as follows: (1) study comparing the effects between robot‐assisted lobectomy and thoracotomy lobectomy; (2) patients treated with any radiotherapy or chemotherapy before the operation; and (3) review, case report, experiment, comment, and letter.

### Literature selection, quality assessment and data extraction

Two reviewers (K.H. and X.T.) independently read the title and abstract of each citation to select eligible studies for further assessment. The full text of potentially eligible citations was retrieved and evaluated for inclusion, and the following information was extracted: (1) first author, publication year, country, patients basic characteristic, language, sample size, study type and median follow‐up time; (2) perioperative outcomes: operation time (minute), blood loss (mL), chest tube duration(day), complication rate, total number of dissected lymph node, and postoperative hospital stay (day); and (3) HRs with 95% CI of OS and DFS were also extracted directly from the text or indirectly calculated from the Kaplan–Meier survival curve from each included study. If the survival data were not directly recorded in primary reports, we calculated HRs with 95% CIs from survival curves as we previous described^29^ and the mean or standard deviation (SD) was estimated according to the recommendation of the Cochrane Collaboration as previous study described if it was not reported directly in the study.^30^ The 9‐star Newcastle‐Ottawa Scale (NOS) was used to assess the methodological quality of cohort study and papers with scores ≥6 were regarded as high quality^31^ and the reliability Jadad scale was used to assess the methodological quality of RCTs, and papers with scores of 4 or more were defined as high quality.^32^

### Statistical analysis

We conducted this meta‐analysis by applying RevMan5.3 software (the Cochrane Collaboration) and STATA 12.0 package (StataCorp) based on the PRISMA guidelines.^28^ For continuous data such as operation time, blood loss, chest tube duration, total number of dissected lymph node, and postoperative hospital stay, the weighted mean differences (WMDs) with 95% confidence intervals (CI) were used for comparison. For categorical data such as complication rate, the risk ratio (RR) with 95% CI was applied. For survival data such as OS or DFS, HRs with 95% CI were used for the quantitative synthesis. To assess the statistical homogeneity among studies, the Cochrane Q‐test and I^2^ statistic were performed, and an I^2^ ≥ 50% or *p* ≤ 0.10 was considered to indicate high heterogeneity. The random‐effect model test (DerSimonian and Laird method) was performed for statistical analysis if high heterogeneity was encountered between studies. Otherwise, the standard fixed‐effect model test (Mantel–Haenszel method) was used for analysis. Sensitivity analysis was performed to estimate the stability of quantitative synthesis results, and the reliability of the meta‐analysis results were robustly confirmed if it did not change significantly.^33^ Subgroup analysis was further conducted based on study design (whether the study applied propensity score matched [PSM] analysis or not). A funnel plot with Egger's tests was used to evaluate publication bias.

## RESULTS

### Literature evaluation

**FIGURE 1:**
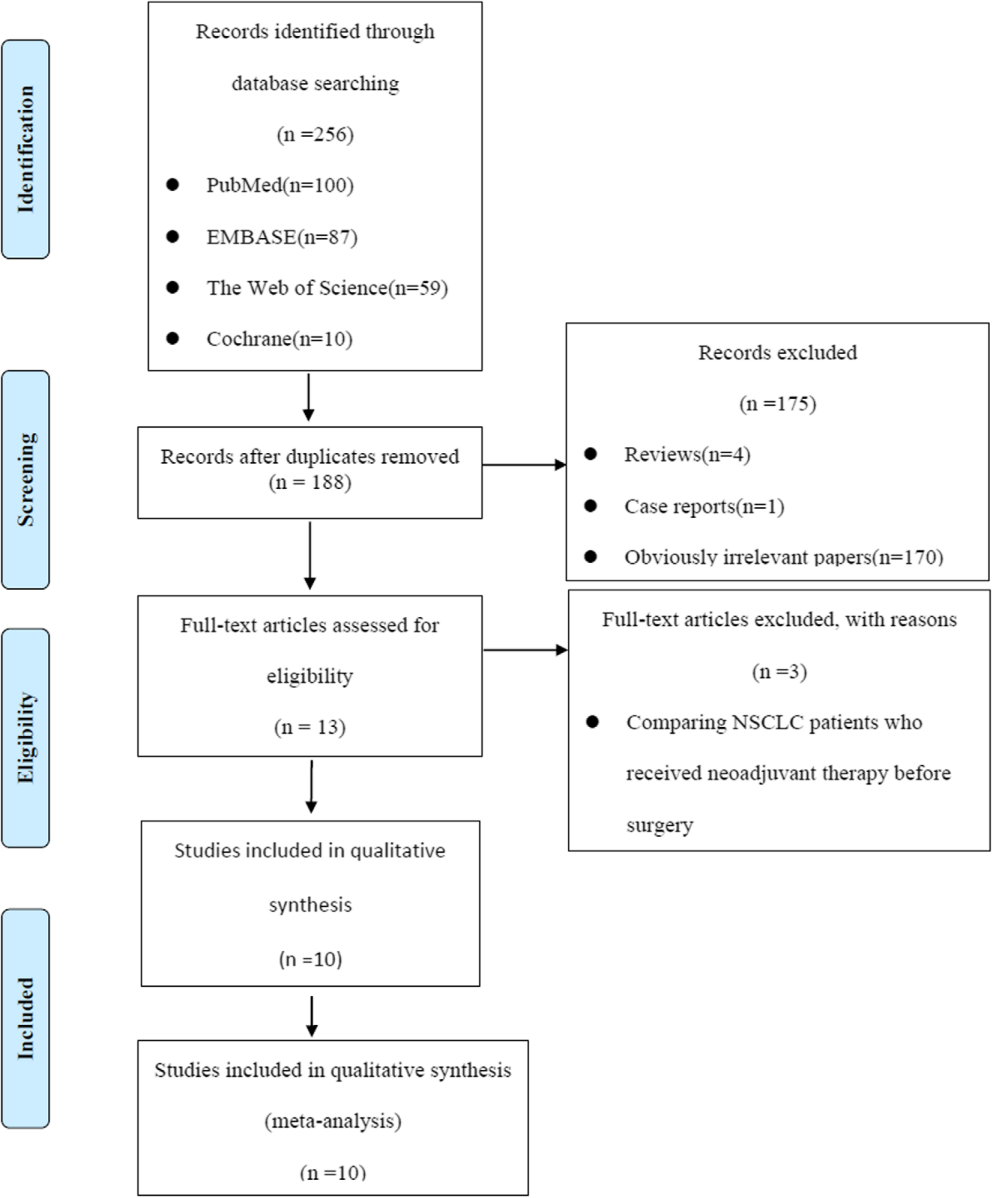
PRISMA flowchart of the relevant literature retrieval. PRISMA, Preferred reporting items for systematic reviews and meta‐analysis

After systematic search from the above database, we identified a total 256 papers. After excluding papers for duplication, 188 papers were reviewed for title and abstract. After removing case reports, reviews, conference abstracts, and non‐relevant papers, a total of 13 papers were carefully reviewed for full‐text and 3 papers focused on comparing NSCLC patients who received neoadjuvant therapy before surgery. Finally, 10 cohort studies with a total 2785 patients were included in this meta‐analysis.^10^, ^14^, ^20^, ^21^, ^22^, ^23^, ^24^, ^25^, ^26^, ^27^ Details of the literature evaluation according to the PRISMA guidelines are shown in Figure 1.

### Study characteristics

All of the 10 included studies were cohort studies. Among them, eight studies were from China and one study was from Japan^20^ and one study was from Korea.^24^ All of the patients included were pathologically diagnosed as NSCLC with N2 mediastinal metastasis intra‐or post‐operative, ranging from IIIA to IIIB, whereas preoperative examination diagnosed as clinical N0. All of them received lobectomy with systemic lymph node dissection (SND) via VATS or thoracotomy straightly except one study, which included some patients who received neoadjuvant therapy before surgery,^14^ however, the percentage of patients receiving neoadjuvant therapy was ~0.93%, which was an extremely small proportion. Therefore, this did not compromise the value of our study and it was included for analysis after careful discussion. Three studies applied PSM method to minimize selection bias and generate well‐balanced groups_,_ ^14^, ^21^, ^26^ and the other studies included patients with comparable baseline characteristics. Therefore, all the included studies had a relatively high quality score ranging from 6 to 8 stars, which indicated a low risk of bias in our meta‐analysis. However, according to the methodology of the Grading of Recommendations Assessment Development and Evaluation (GRADE)^34^ for evaluating the quality of the evidence presented in the meta‐analysis, all these included studies were classified as low‐quality evidence (i.e., permitting low confidence in the estimated effects). The detailed characteristics of the included studies were shown in Table 1.

**TABLE 1:** Characteristics of included studies

Study	Country	Patients	Study type	Sample size			Surgical type		Follow up (month)	Neoadjuvant therapy (%)	Stage	Quality scores
				Total	VATS	Thoracotomy	VATS	Thoracotomy				
Wang et al.^22^	China	Patients with early stage NSCLC before the operation (cT1‐2N0M0, stage I), but found to have unexpected minimal mediastinal lymph node metastasis post‐operatively	Cohort study	320	101	219	Lobectomy with SND		37	No	IIIA	8
Zhong et al.^10^	China	Patients with clinical cT1‐2aN0M0, but found to have unexpected minimal mediastinal metastasis post‐operatively	Cohort study	157	67	90	Lobectomy with SND		35.6	No	IIIA	7
Li and Wang^26^	China	NSCLC patients who underwent VATS or open thoracotomy with clinical N0, but postoperatively pathological N2	Cohort study	76	29	47	Lobectomy with SND		16 months in VATS group and 72 months in thoracotomy group	No	IIIA‐IIIB	8
Yoohwa et al.^24^	Korea	NSCLC patients who underwent curative anatomical resection and systemic mediastinal lymph node dissection for pN2 by thoracoscopic surgery or thoracotomy	Cohort study	536	95	441	Lobectomy with SND		72	NA	NA	6
Watanabe et al.^20^	Japan	Patients with clinical N0 and pathological N2 NSCLC (cN0‐pN2 NSCLC)	Cohort study	69	37	32	Lobectomy with SND		NA	No	IIIA‐IIIB	7
Liu et al.^14^	China	NSCLC patients with pathological N2 and were divided into VTAS group and thoracotomy group through propensity score matching (PSM) method.	Cohort study	592	296	296	Lobectomy with SND		36	Yes(4/100)	IIIA‐IIIB	8

### Comparison of short‐term outcomes between VATS group and thoracotomy group

**FIGURE 2:**
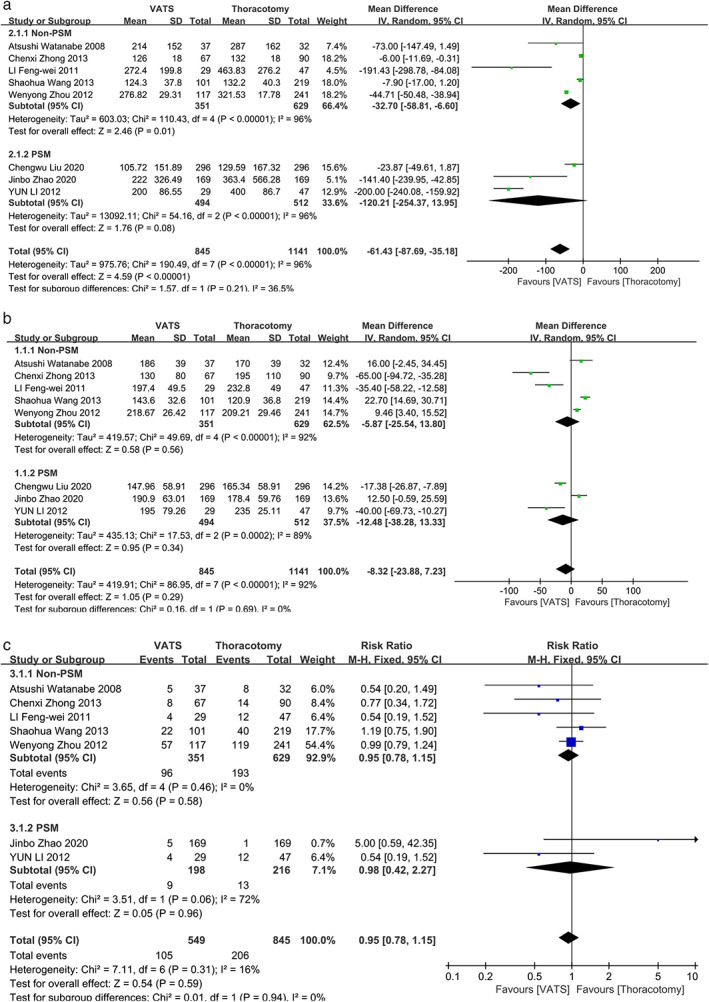
Forest plots of (a) blood loss, (b) operation time, and (c) post‐operation complication rate; PSM, propensity score matched; VATS, video‐assisted thoracoscopic surgery; CI, confidence interval

**FIGURE 3:**
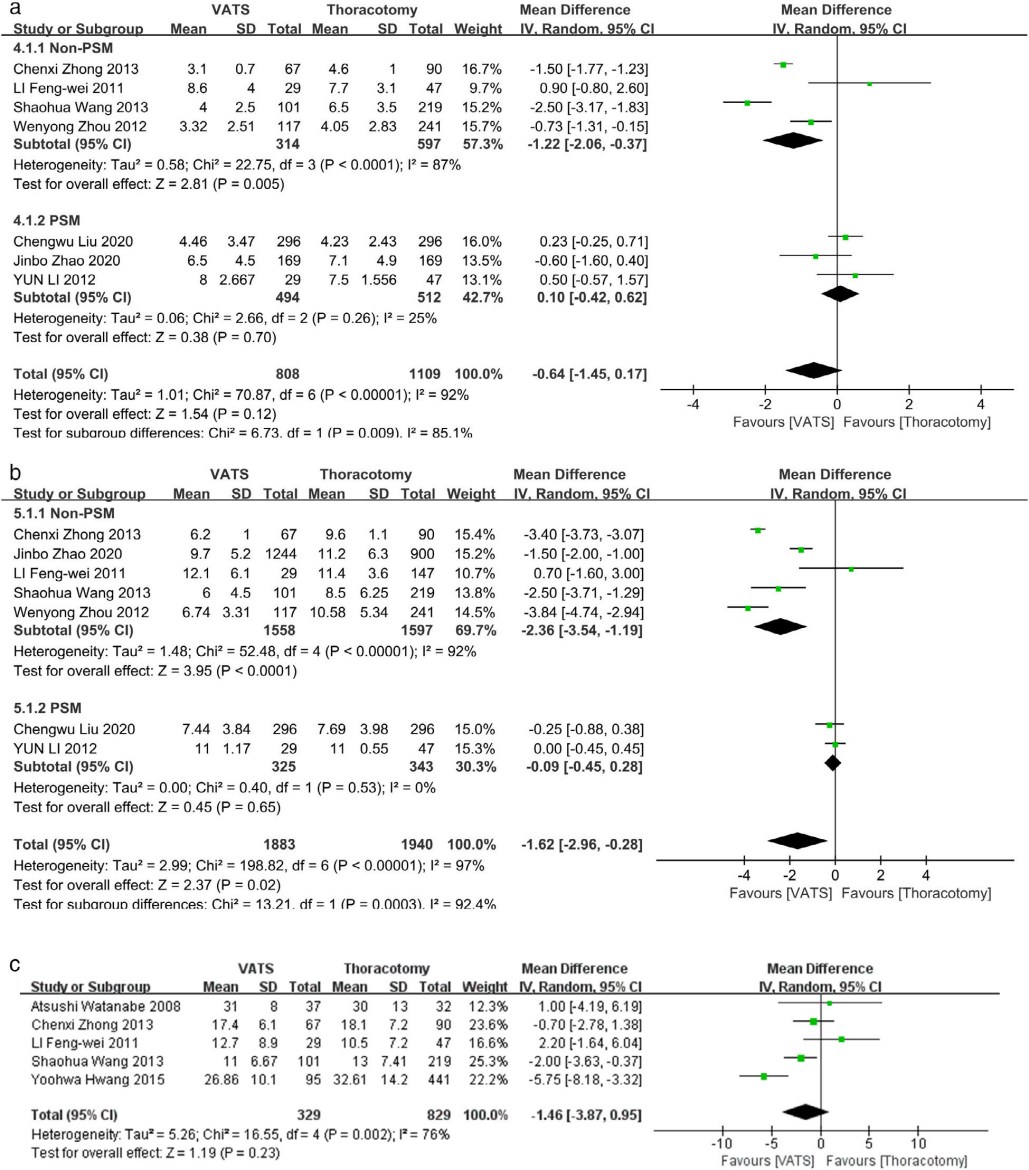
Forest plot of (a) chest tube duration, (b) post‐operation hospital stay and (c) total number of dissected lymph node; PSM, propensity score matched; VATS, video‐assisted thoracoscopic surgery; CI, confidence interval

Eight studies reported the results of operation time, blood loss and complication rate.^10^, ^14^, ^20^, ^21^, ^22^, ^25^, ^26^, ^27^ The results showed that VATS for NSCLC patients with pN2 disease yielded significantly less blood loss (WMD, −61.43; 95% CI, [−87.69, −35.18]; *p* < 0.001) than those receiving thoracotomy (Figure 2(a)). Moreover, VATS surgery could yield comparable operation time (WMD, −8.32; 95% CI, [−23.88, 7.23]; *p* = 0.29) and post‐operation complication rate (RR, 0.95; 95% CI, [0.78, 1.15]; *p* = 0.59) to thoracotomy (Figure 2(b),(c)). Seven studies reported the results of chest tube duration and post‐operative hospital stay.^10^, ^14^, ^21^, ^22^, ^23^, ^26^, ^27^ VATS surgery could yield comparable chest tube duration to thoracotomy (WMD, −0.64; 95% CI, [−1.45, 0.17]; *p* = 0.12) (Figure 3(a)) with significantly less post‐operation hospital stay (WMD, −1.62; 95% CI, [−2.96, −0.28]; *p* = 0.02) (Figure 3(b)). As for SND, five studies reported the total number of dissected lymph node^10^, ^20^, ^22^, ^24^, ^27^ and the result indicated that VATS could yield comparable extent of lymph node dissection to thoracotomy (WMD, −1.46; 95% CI, [−3.87, 0.95]; *p* = 0.23) (Figure 3(c)).

### Comparison of long‐term outcomes between VATS group and thoracotomy group

**FIGURE 4:**
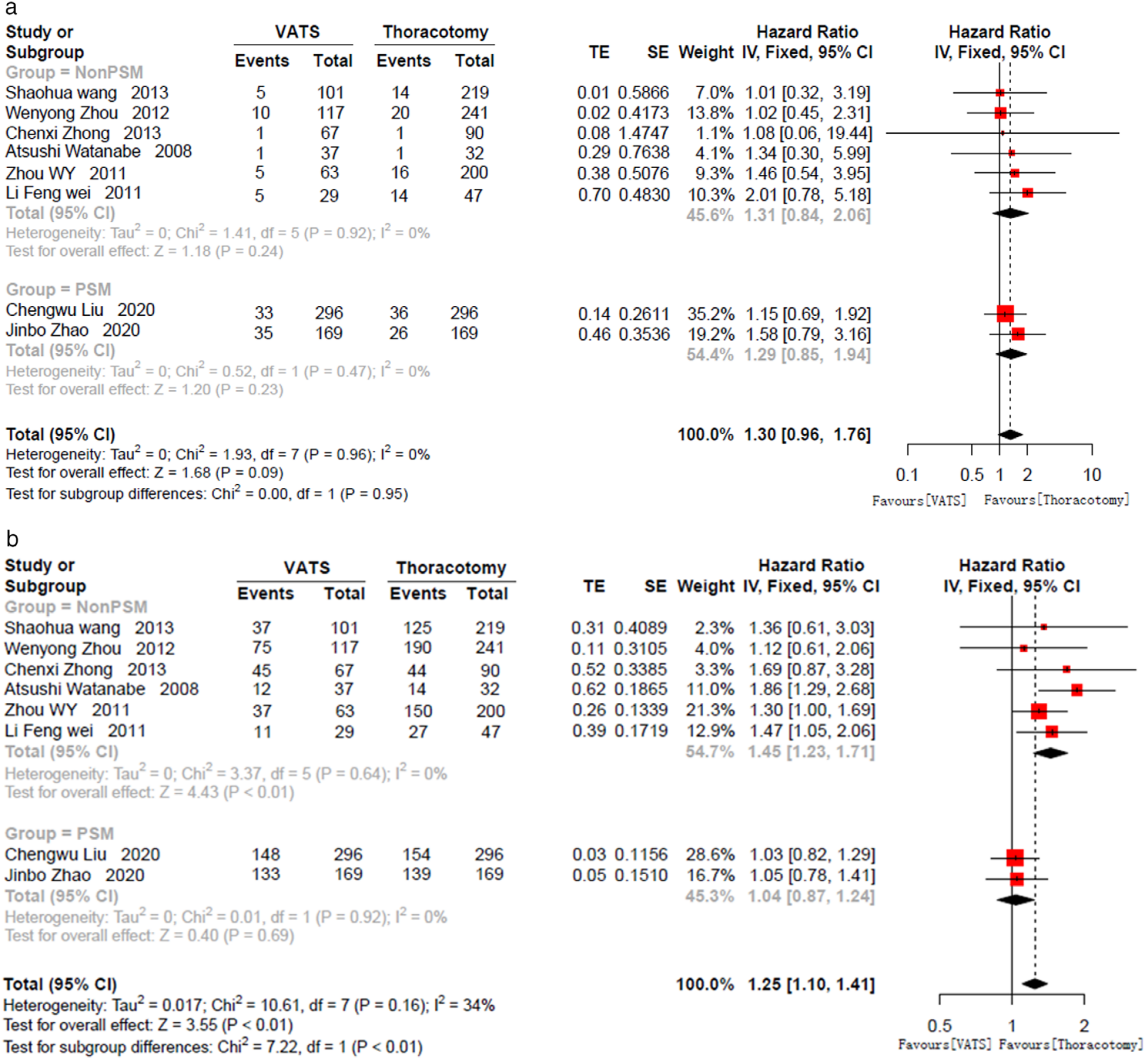
Forest plot of (a) 1‐year OS, and (b) 3‐year OS; PSM, propensity score matched; VATS, video‐assisted thoracoscopic surgery; OS, overall survival; CI, confidence interval

**FIGURE 5:**
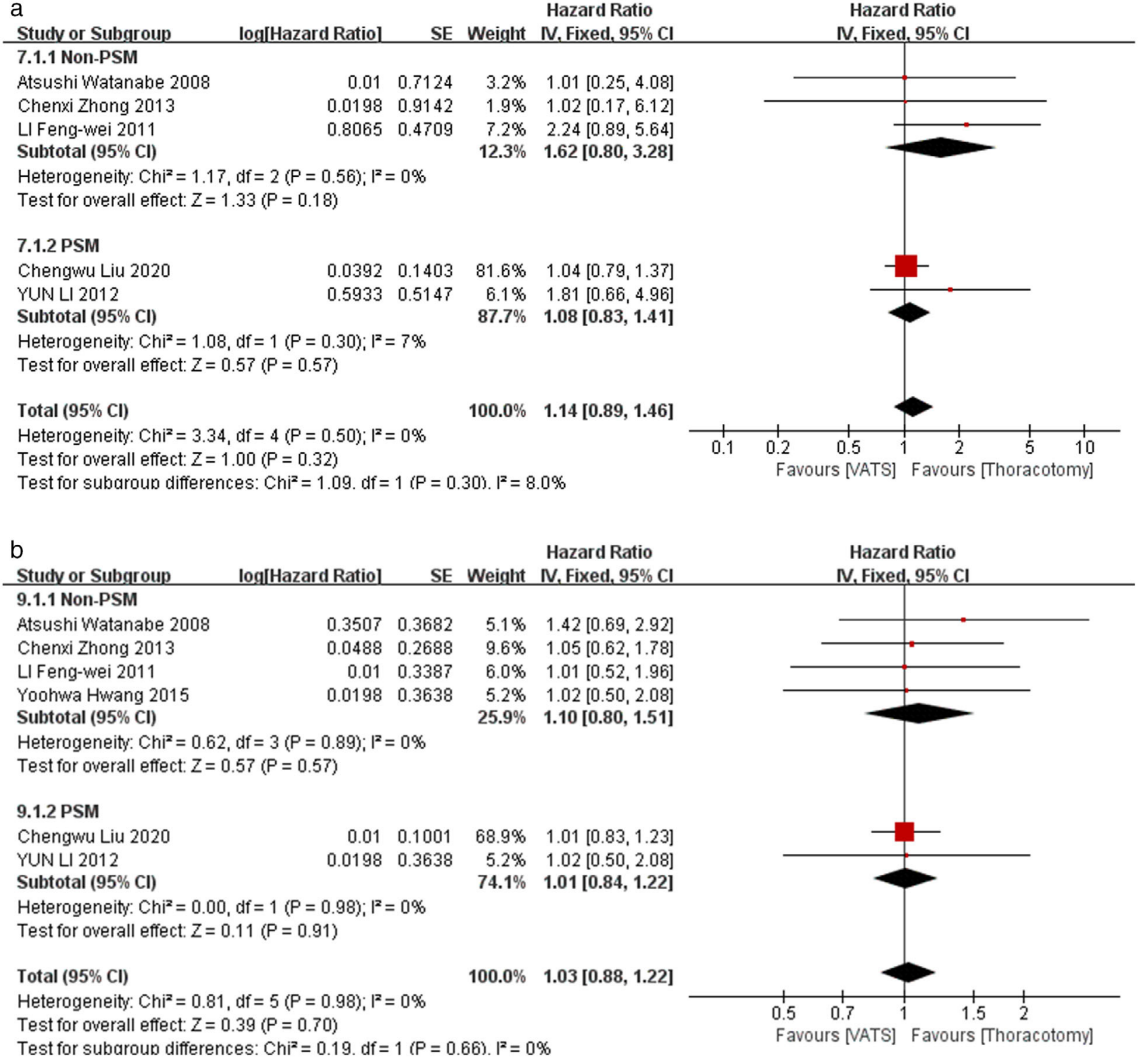
Forest plot of (a) 1‐year DFS, and (b) 3‐year DFS; PSM, propensity score matched; VATS, video‐assisted thoracoscopic surgery; DFS, disease‐free survival; CI, confidence interval

HRs and 95% CI of OS and DFS were extracted and calculated from the included studies.^10^, ^14^, ^20^, ^21^, ^22^, ^23^, ^24^, ^25^, ^27^ The data of 1‐year and 3‐year OS could be extracted and calculated from eight of them^10^, ^14^, ^20^, ^21^, ^22^, ^23^, ^25^, ^27^ through the methods as we previously reported.^29^ However, the data of 1‐year and 3‐year DFS could only be extracted and calculated from five studies^10^, ^14^, ^20^, ^26^, ^27^ and six studies^10^, ^14^, ^20^, ^24^, ^26^, ^27^, respectively. Our meta‐analysis showed that VATS surgery could yield comparable 1‐year OS (HR, 1.30; 95% CI, [0.96, 1.76]; *p* = 0.09) (Figure 4(a)) and better 3‐year OS (HR, 1.26; 95% CI, [1.12, 1.42]; *p* = 0.0002) for pN2 NSCLC patients (Figure 4(b)). However, the 1‐year DFS (HR, 1.14; 95% CI, [0.89, 1.46]; *p* = 0.32) and 3‐year DFS (HR, 1.03; 95% CI, [0.88, 1.22]; *p* = 0.70) were comparable between VTAS group and thoracotomy group (Figure 5(a),(b)).

### Subgroup analysis based on studies with PSM analysis

Three studies applied PSM analysis to balance the variables that might influence the outcomes between VATS group and thoracotomy group.^14^, ^21^, ^26^ Therefore, we further conducted subgroup meta‐analysis by only including these studies. Our results indicated that VATS surgery could still yield comparable operation time (Figure 2(b)), post‐operation complication rate (Figure 2(c)) and chest tube duration time (Figure 3(a)) as well as 1‐year DFS (Figure 5(a)) and 3‐year DFS (Figure 5(b)) to thoracotomy. However, in the analysis of PSM studies, VATS surgery yielded similar blood loss (Figure 2(a)), post‐operation hospital stay (Figure 3(b)) as well as 1‐year OS (Figure 4(a)) and 3‐year OS (Figure 4(b)).

### Sensitivity analysis and publication bias

Sensitivity analysis was further performed to assess the stability of the summarized estimates of 3‐year OS and post‐operation complication rate as well as total number of dissected lymph node. The sensitivity analysis showed that the overall results of 3‐year OS (Figure S1(a)), post‐operation complication rate (Figure S1(b)), and total number of dissected lymph node (Figure S1(c)) did not change after the sequential removal of each study, indicating that the results of our meta‐analysis had a relatively high stability because no substantial heterogeneity was found between the adjusted estimates and primary estimates. Moreover, as the funnel plot demonstrated, there was no asymmetry associated with publication bias based on a visual inspection and the Egger's tests of 3‐year OS (*p* = 0.184) (Figure S2(a)), post‐operation complication rate (*p* = 0.578) (Figure S2(b)), and total number of dissected lymph node (*p* = 0.538) (Figure S2(c)) also showed no significant potential publication bias among these studies.

## DISCUSSION

VATS lung surgery has been widely adopted to treat early stage lung cancer by thoracic surgeons since it was introduced at the beginning of the 1990s^16^ with its significant safety and feasibility compared to traditional thoracotomy.^17^ However, for cN0‐pN2 NSCLC patients, it remains controversial whether VTAS is effective in lymph node dissection and whether it is necessary to convert VATS to thoracotomy when intraoperative LN sampling reveals pathologic N2 disease.^19^ Extensive attention has been paid to whether the VATS lobectomy has at least comparable long‐term oncology efficacy to thoracotomy.^22^ Therefore, we conducted this up‐to‐date systematic review and meta‐analysis to justify for the application of VATS lobectomy for cN0‐pN2 NCSLC.

In this meta‐analysis, we include a total of 10 cohort studies with a total of 2785 NSCLC patients. We found that VATS surgery could yield significantly less blood loss and shorter postoperative hospital stay than thoracotomy lobectomy, and there was no significant difference of operation time, postoperative complication rate, chest tube duration, total number of dissected lymph node, as well as 1‐year DFS and 3‐year DFS between VATS lobectomy and thoracotomy lobectomy. Moreover, VATS lobectomy may yield a comparable 1‐year OS and better 3‐year OS compared with thoracotomy lobectomy. However, in the analysis of well‐matched studies with PSM, VATS lobectomy could also yield comparable operation time, blood loss, chest tube duration, postoperative hospital stay, postoperative complication rate, and there was no significant difference of 1‐year OS, 3‐year OS as well as 1‐year DFS and 3‐year DFS. Therefore, our results indicated that VATS lobectomy is a feasible approach to management of cN0‐pN2 NSCLC without compromising the chance of curability. It is unnecessary to convert the VATS approach to thoracotomy even if intraoperative LN sampling reveals pathologic N2 disease. Although our study showed that VATS for NSCLC patients with pN2 disease yielded significantly less blood loss, less post‐operation hospital stay, comparable operation time, comparable chest tube duration, and post‐operation complication rate than those receiving thoracotomy, the inter‐study heterogeneity is inevitable because of many confounding factors, such as patient selection criteria, surgical methods and quality, surgery volume as well as surgeon's experience. All these factors could have an influence on the short‐time outcomes comparison between VATS group and thoracotomy group.

Previous studies have demonstrated that VATS lobectomy was associated with superior short‐term outcomes and non‐inferior oncological outcomes compared with thoracotomy for early‐stage NSCLC.^17^ Our meta‐analysis also showed that VATS approach could achieve comparable short‐term and long‐term clinical outcomes to thoracotomy for cN0‐pN2 NSCLC. For NSCLC patients with mediastinal lymph node metastasis, several factors including inadequate experience during training,^35^ narrow visual field with limited range of instrumental movement,^36^ and inferior oncological effectiveness (e.g., lymph node assessment) may contribute to the major concerns of VATS for pN2 NSCLC.^21^, ^37^ However, Li et al.^26^ performed a retrospective analysis of 76 patients comparing clinical outcomes for patients with clinical N0, but pathologic N2 NSCLC patients after VATS lobectomy and thoracotomy lobectomy, they found that VATS approach could yield similar total number of dissected mediastinal lymph node and the total number of dissected lymph node stations. Zhong et al.^10^ also found the similar result that VATS approach was effective in lymph node dissection compared with thoracotomy. Sagawa et al.^38^ performed VATS lobectomy and lymph node dissection followed by open thoracotomy check‐up and found only 2% to 3% of lymph nodes remnant. Hoksch et al.^39^ performed systematic lymph node dissection on 13 corpses by VATS, followed by immediate open thoracotomy to evaluate the dissection effect and he found no bronchial or mediastinal lymph nodes remnant. In this meta‐analysis, we also showed that VATS approach was feasible in lymph node dissection compared with thoracotomy for cN0‐pN2 NSCLC; therefore, there is no need to convert the VATS approach to thoracotomy when intraoperative LN sampling reveals pathologic N2 disease. Moreover, our result indicated that VATS approach tended to yield a better long‐term oncology outcome for pN2 NSCLC, which still held true in the analysis of well‐matched studies with PSM. Previous study demonstrated that VATS approach caused less trauma and had little influence on immune system; therefore, its ability to prevent complication and reduce residual cancer cells after surgery was more effective than thoracotomy.^40^ In addition, patients with VATS recovered quickly, and therefore, the interval between surgery and the first adjuvant treatment was shorter for them as compared to those receiving thoracotomy.^26^ Moreover, a better condition for administration of adjuvant therapy after VATS approach can be created and it is possible that proliferation from micro‐metastasis of tumor cells, which exists prior or related to surgery, and the fixed value occurrence probability, can be reduced through shortening the so‐called post‐surgery treatment waiting time.^23^ All these factors combined together may help explain why patients receiving VATS lobectomy have a better survival tendency than thoracotomy. Zhou et al.^23^ studied the oncology outcomes of VATS lobectomy for unexpected pN2 NSCLC and found that patients in VATS group had a better overall survival time and survival rates at one, three and 5 years after surgery. Another reported by Wang et al.^22^ also indicated that patients in VATS group had a better overall survival time than those in thoracotomy group. Taken together, we believe that the minimally invasive surgery through VATS lobectomy with lymph node dissection is feasible and safe for unexpected pN2 NSCLC with at least comparable long‐term oncology outcomes to traditional thoracotomy lobectomy and there is no need to convert the VATS approach to thoracotomy even if intraoperative LN sampling reveals pathologic N2 disease. To the best of our knowledge, this is the most comprehensive systematic review and meta‐analysis focusing on this topic.

## LIMITATIONS OF THE STUDY

Our study had several limitations. First, all the included studies were retrospective cohort studies with relatively low quality evidence, therefore, the quality and validity of our meta‐analysis was decreased. Second, patient selection bias existed when VATS lobectomy and thoracotomy lobectomy were retrospectively compared and only three papers applied PSM analysis. Third, most of these studies were carried out from China and it needs further investigation whether similar conclusion would hold in Western countries. Fourth, the sample size of the studies included in our meta‐analysis was relatively small, and the final pathological stage varied among studies. Moreover, we failed to conduct subgroup analysis based on tumor stage because most of the included studies did not perform further subgroup analysis according to pathological stage. Fifth, two studies by Wang et al.^22^ and Zhou et al.^23^ might select and analyze some overlapping patients with pN2 NSCLC in the same department during different periods, which could have an influence on the conclusion. Furthermore, the details in lymph node clearance including the comparison of the number of dissected N1 lymph nodes, N2 lymph nodes, N1 stations, and N2 stations between the two surgical approaches failed to be comprehensively presented and analyzed, because there were no papers to analyze these detailed data, respectively, between VATS group and thoracotomy group. Finally, studies included in this meta‐analysis were based on NSCLC patients who were diagnosed as clinical N0 before surgery, but found to have unexpected mediastinal lymph node metastasis intra‐ or post‐operatively pN2 with single or multiple station metastasis. However, we failed to further conduct analysis based on mediastinal lymph node metastasis stations, which could affect the oncological outcomes alone rather than surgery approach.^22^, ^23^ Therefore, further RCTs with rigorous and appropriate adjustments are urgently needed to confirm and update our conclusions.

## CONCLUSION

We performed the most comprehensive systematic review and meta‐analysis comparing the feasibility and oncological outcomes of VATS versus thoracotomy for NSCLC patients with pN2 disease. VATS approach could yield comparable extent of lymph node dissection with less surgical trauma to thoracotomy and may improve post‐operative recovery. Moreover, it may improve the oncological outcomes of those patients. Therefore, VATS could be recommended as an alternative to thoracotomy for treating pN2 NSCLC in carefully selected cases and it is unnecessary to convert the VATS approach to thoracotomy to complete SND even if pN2 disease is revealed intraoperatively. Further large‐scale, multicenter well‐conducted RCTs, however, are needed to confirm and update our conclusions.

### STATEMENTS AND DECLARATIONS

## CONFLICT OF INTERESTS

The authors declare no competing interests.

## ETHICAL APPROVAL

Ethical approval is not required for this type of studies (systematic review).

## INFORMED CONSENT

Informed consent does not apply for this type of studies (systematic review).

## Supporting information


**Appendix S1** Supporting Information
