# Supplementary material for: Feasibility and oncological outcomes of video‐assisted thoracic surgery versus thoracotomy for pathologic N2 disease in non–small cell lung cancer: A comprehensive systematic review and meta‐analysis
Source: Thorac Cancer. 2022 Sep 14;13(21):2917–28. doi: 10.1111/1759-7714.14614 (PMC9626309; doi:10.1111/1759-7714.14614)
Supplement: Supplementary file 1 — Appendix S1 Supporting Information [file TCA-13-2917-s001.pptx]

## Slide 1
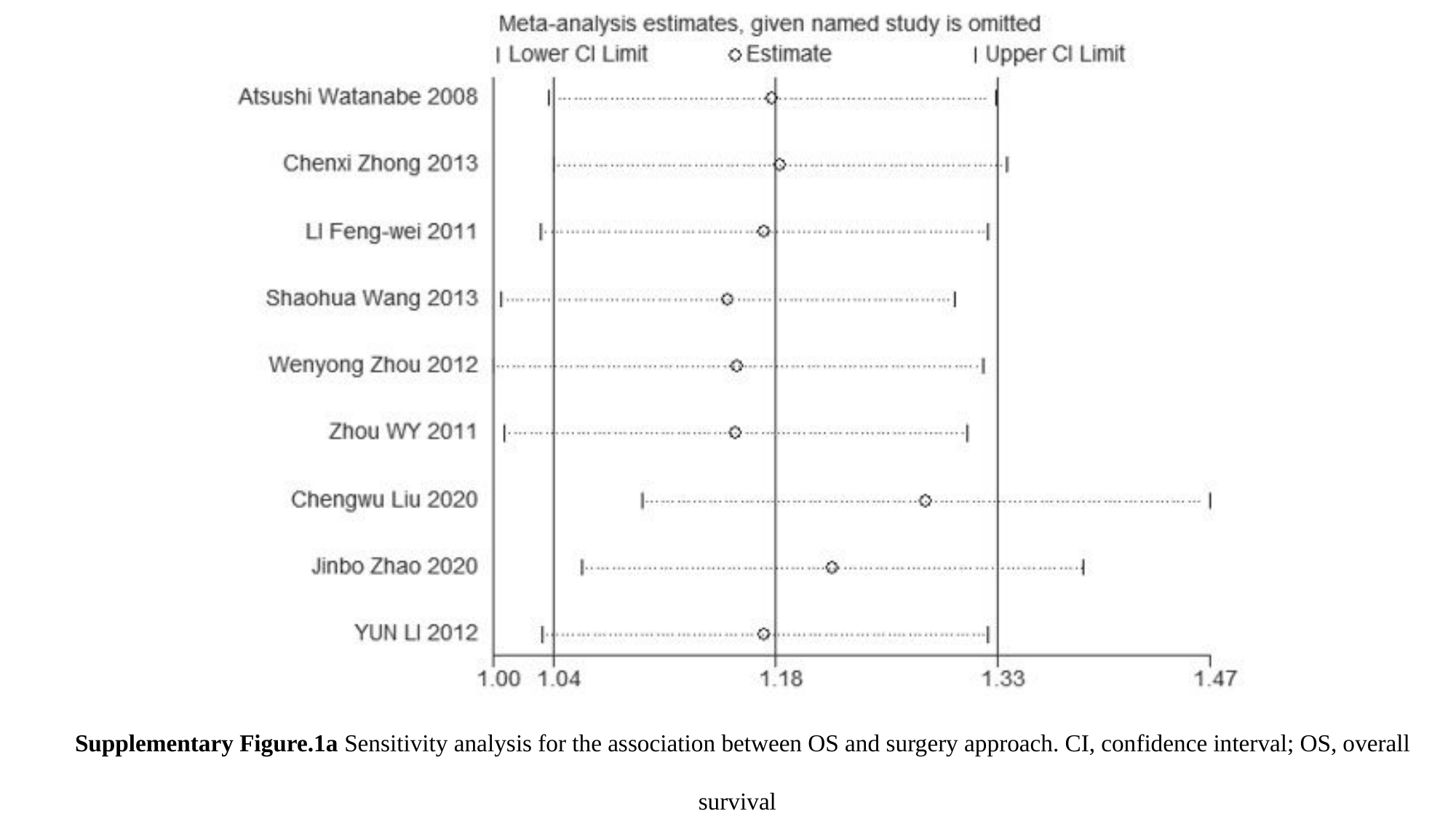

Supplementary Figure.1a Sensitivity analysis for the association between OS and surgery approach. CI, confidence interval; OS, overall survival

## Slide 2
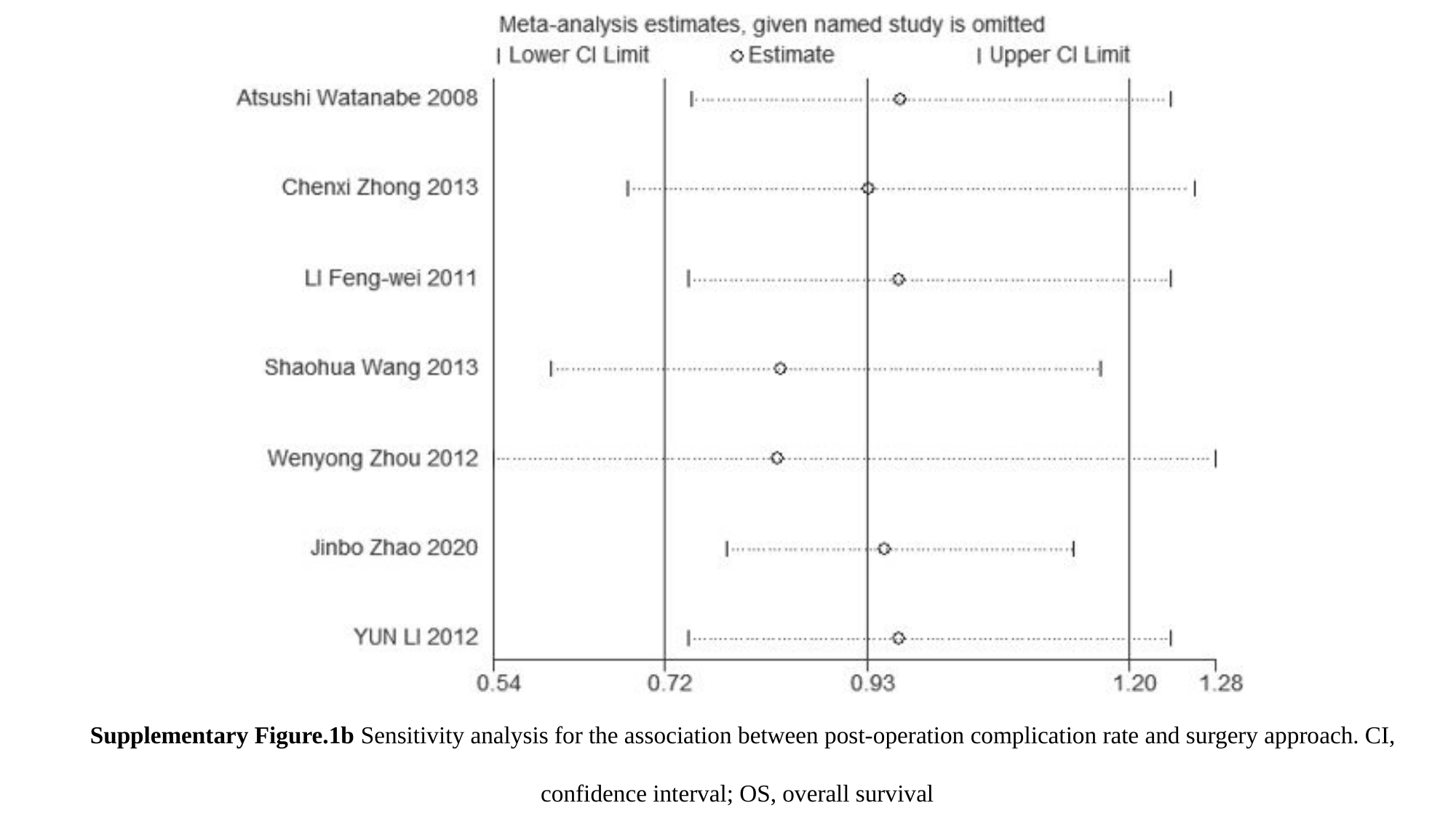

Supplementary Figure.1b Sensitivity analysis for the association between post-operation complication rate and surgery approach. CI, confidence interval; OS, overall survival

## Slide 3
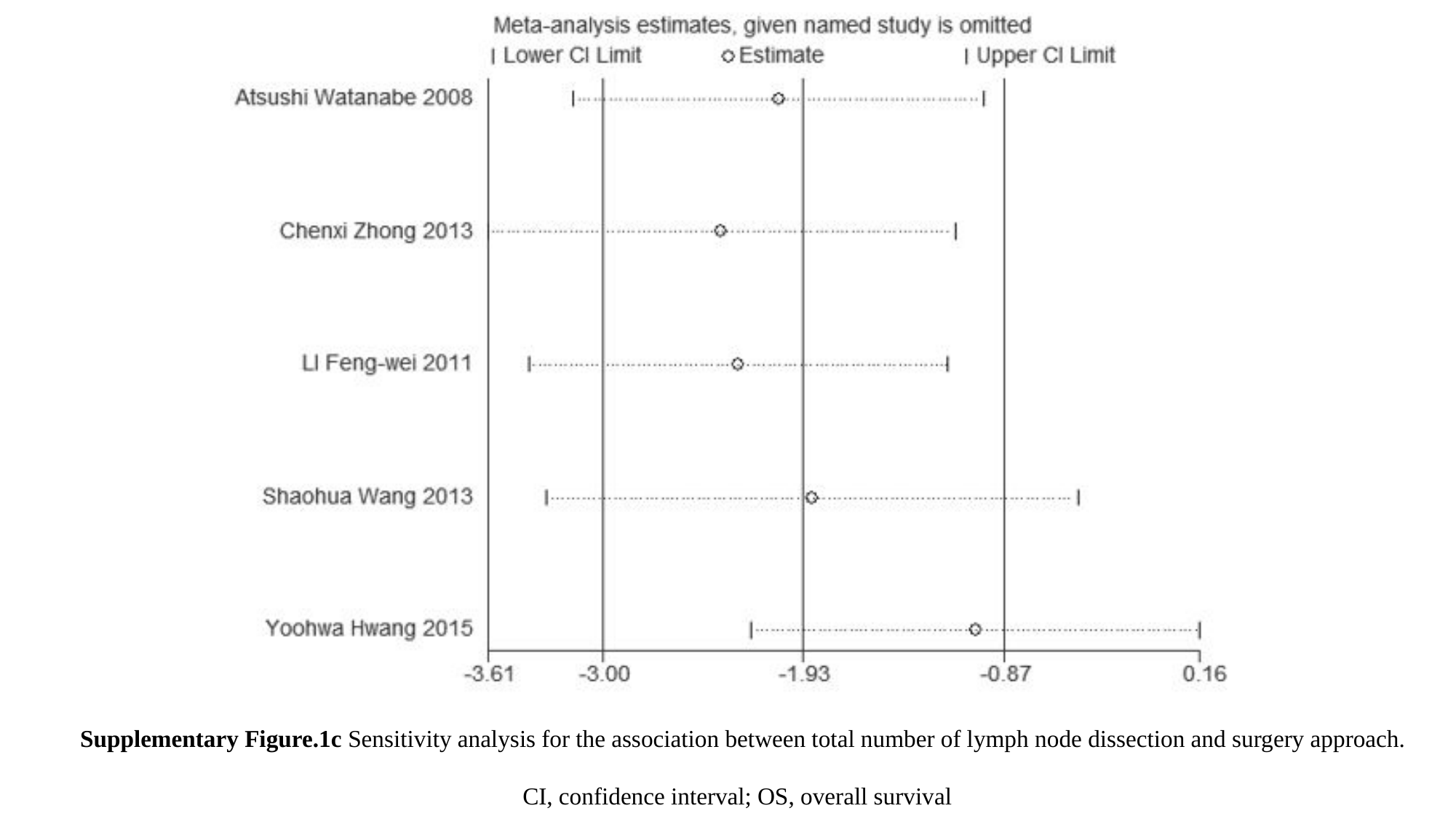

Supplementary Figure.1c Sensitivity analysis for the association between total number of lymph node dissection and surgery approach. CI, confidence interval; OS, overall survival

## Slide 4
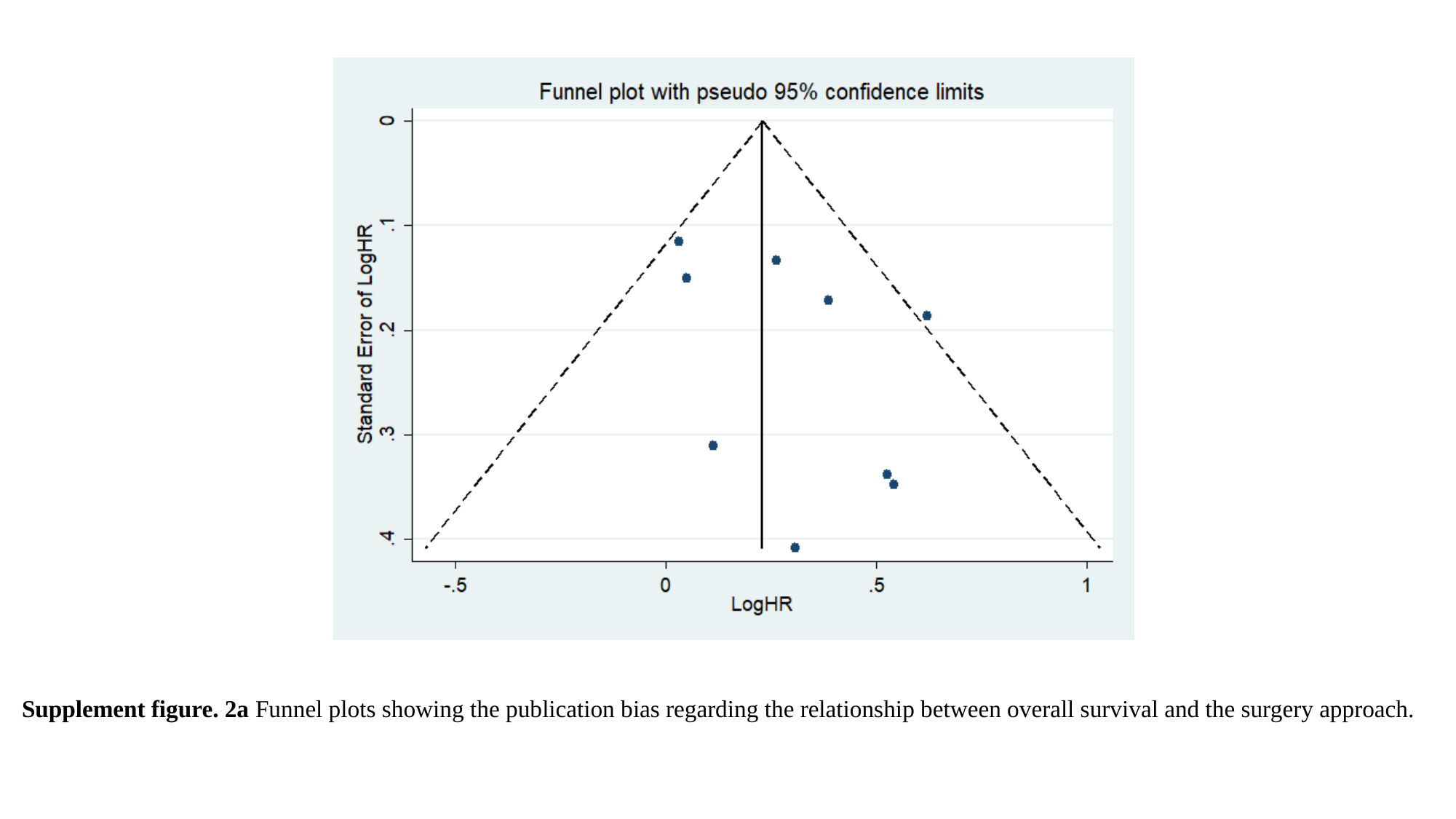

Supplement figure. 2a Funnel plots showing the publication bias regarding the relationship between overall survival and the surgery approach.

## Slide 5
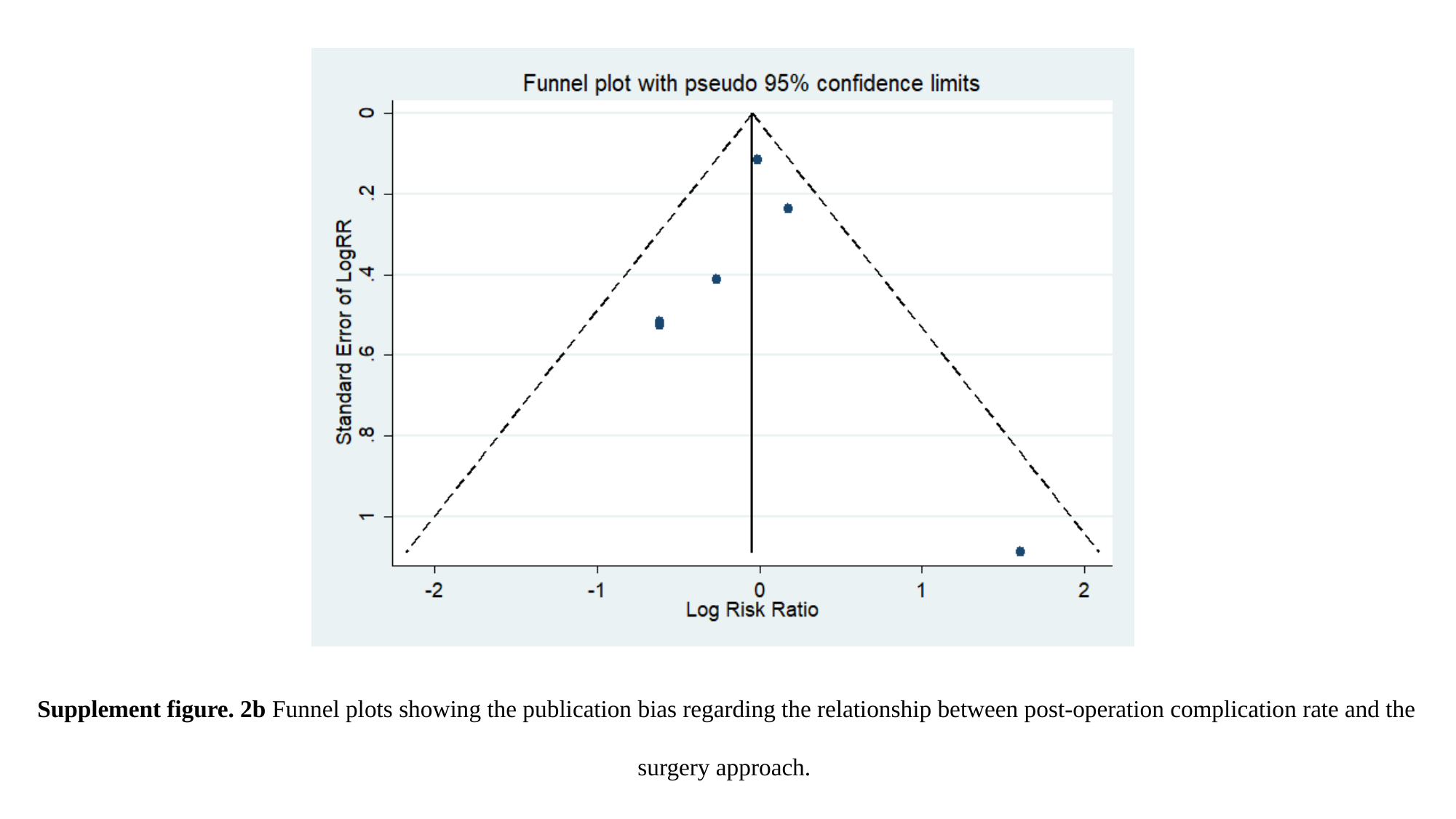

Supplement figure. 2b Funnel plots showing the publication bias regarding the relationship between post-operation complication rate and the surgery approach.

## Slide 6
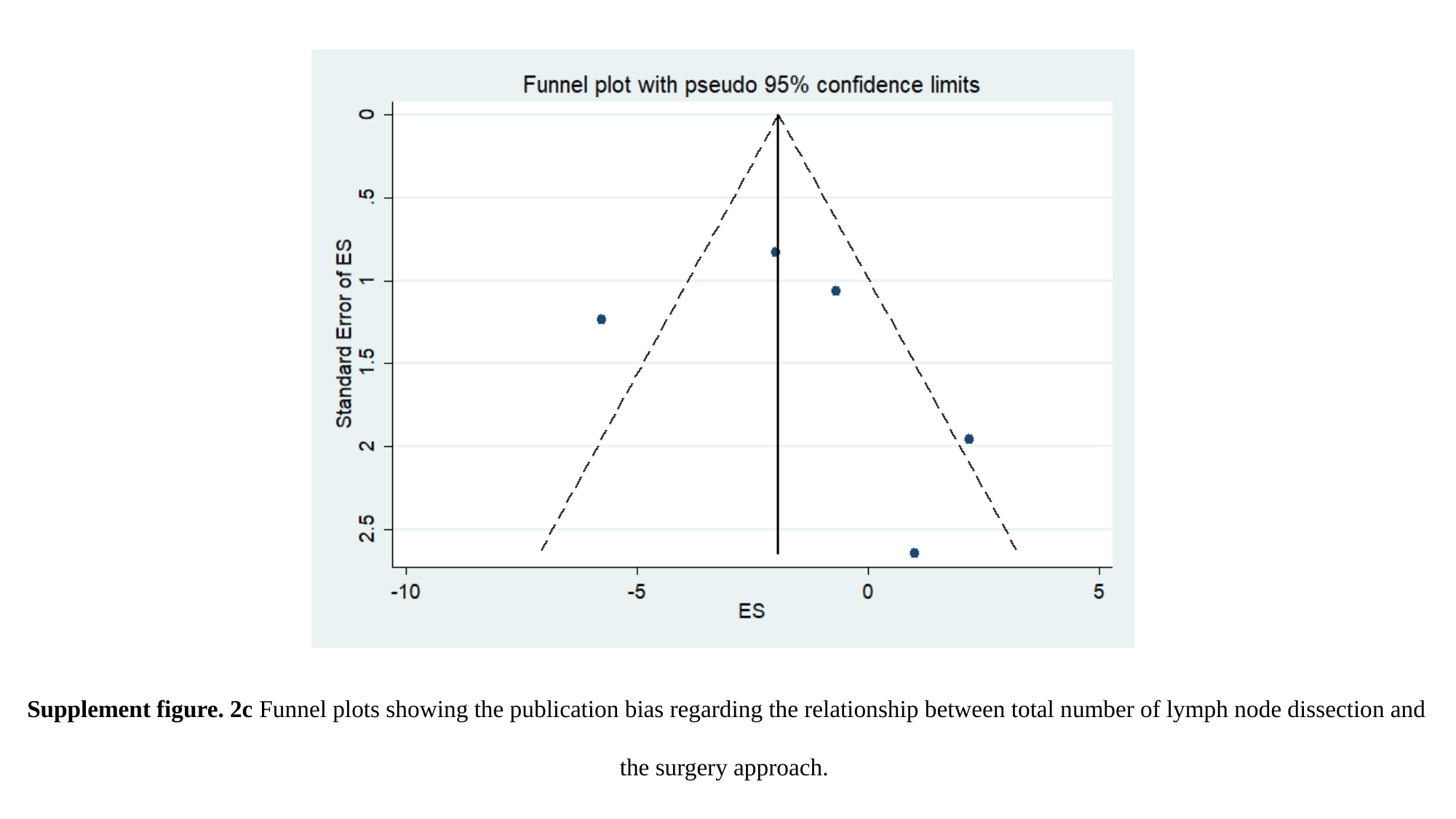

Supplement figure. 2c Funnel plots showing the publication bias regarding the relationship between total number of lymph node dissection and the surgery approach.
